# The State Medical Society of Pennsylvania

**Published:** 1860-05

**Authors:** 


THE STATE MEDICAL SOCIETY OF PENNSYLVANIA will hold its annual meeting in this city on the second Wednesday in June.

